# Abstract of Meteorological Observations for Deeember, 1853, Made at Philadelphia, Pa.

**Published:** 1854-02

**Authors:** James A. Kirkpatrick


*Abstract of Meteorological Observations for December,* 1853, *made at Philadelphia, Pa. By* PROF. JAMES A. KIRKPATRICK.

| 1853. Dec. | BAROMETER. Corrected for Temperature. Mean. | Range. | THERMOMETER. Mean. | Range. | Dew point 2 P. M. | Prevailing Winds. | General Remarks. |
|---|---|---|---|---|---|---|---|
| | Inches. | Inch. | deg. | deg. | deg. | | |
| 1 | 29.969 | .045 | 40.00 | 6 | 29.0 | N.N.W. | Morn. clear, aft. and ev. cloudy. |
| 2 | 29.957 | .052 | 40.25 | 2 | 33.0 | N.E. | Cloudy. *Rain* 5½ to 9 P.M. |
| 3 | 29.996 | .020 | 39.00 | 6 | 36. | W.N.W. | Cloudy. |
| 4 | 30.186 | .075 | 32.50 | 7 | 24.3 | N.W. | Clear. |
| 5 | 30.216 | .047 | 34.00 | 10 | 29.7 | N.E. | M. & af. cloudy, ev. clear; *Snow* 9 |
| 6 | 29.916 | .262 | 42.50 | 27 | 46.7 | S.W. | do. do. ev. do. [to 10 A.M |
| 7 | 30.043 | .174 | 38.50 | 13 | 31. | N.W. | do. do. ev. do. |
| 8 | 30.112 | .045 | 29.50 | 7 | 26. | (Var.) | Clear. |
| 9 | 30.023 | .070 | 31.00 | 18 | 26. | (Var.) | do. |
| 10 | 29.881 | .020 | 43.00 | 18 | 26.3 | N. | Morn. cloudy, aft. and ev. clear. |
| 11 | 29.897 | .032 | 44.00 | 18 | 32. | N.W. | Clear. |
| 12 | 29.960 | .074 | 43.00 | 14 | 36. | N.W. | M. and ev. clear, aft. cloudy. |
| 13 | 29.969 | .082 | 39.50 | 11 | 33.3 | N.N.E. | M. and aft. cloudy, ev. clear. |
| 14 | 30.012 | .057 | 36.50 | 11 | 32.7 | N. | Clear. |
| 15 | 29.938 | .074 | 36.50 | 13 | 31.3 | N.N.W. | do. |
| 16 | 29.848 | .142 | 33.50 | 5 | 33.7 | S.W.,N.W. | M. cloudy, aft. and. ev. foggy. |
| 17 | 29.436 | .555 | 39.00 | 10 | 39.3 | E.N.E. | M. fog; cl'y; *R.* 11 A.M. to 10 P.M. |
| 18 | 29.418 | .549 | 35.00 | 15 | 27. | NW,W,SW | M. and aft. cloudy, ev. clear. |
| 19 | 29.756 | .061 | 31.75 | 8½ | 26.7 | S.E. | Cloudy, 4½ P.M. *S.* stop dur. night |
| 20 | 30.134 | .231 | 24.00 | 10 | 24.3 | N.W. | M. and ev. clear, *S.* 2½ to 3 P.M. |
| 21 | 30.199 | .139 | 22.50 | 9 | 20. | S.W. | M. and ev. clear, aft. cloudy. |
| 22 | 30.098 | .097 | 32.00 | 14 | 29.7 | S.W. | M. and aft. cloudy, ev. clear. |
| 23 | 29.603 | .182 | 32.00 | 20 | 42. | W.N. | M. foggy, aft. cloudy, ev. clear. |
| 24 | 29.854 | .021 | 25.00 | 8 | 22. | W. | Clear. |
| 25 | 29.867 | .021 | 28.00 | 12 | 29.3 | S.W. | do. |
| 26 | 29.473 | .130 | 35.00 | 14 | 28.0 | SW,WNW | M. and ev. clear, aft. cloudy. |
| 27 | 29.701 | .140 | 32.00 | 8 | 22. | N.W. | do. do. do. |
| 28 | 29.607 | .307 | 35.50 | 15 | 33.7 | (Var.) | Cl'y; *R.* 5 P.M. soon changed to *S.* |
| 29 | 29.509 | .310 | 19.00 | 8 | 16. | N.W. | M. cloudy, ev. clear. [strong w. |
| 30 | 29.651 | .223 | 22.50 | 23 | 23. | E.S.W. | Cloudy, *S.* storm 11 A.M. to 6 P.M. |
| 31 | 29.705 | .088 | 31.50 | 11 | 34.7 | (Var.) | do. *S.* 9½ P.M, stop dur. night. |
| Mean | 29.869 | | 33.81 | | 29.83 | N.W. | Rain and melted snow during the month 2.165 inches. |
| High. | 30.288 | on 21st | 56° on 6th. | | 46.7 on 6th. | | |
| Low. | 29.115 | on 17th | 11° on 30th. | | 16.0 on 29th. | | |
| range | 1.173 inch. | | 45° | | 30.7° | | |

